# Phytochemicals from *Camellia nitidissima* Chi Flowers Reduce the Pyocyanin Production and Motility of *Pseudomonas aeruginosa* PAO1

**DOI:** 10.3389/fmicb.2017.02640

**Published:** 2018-01-09

**Authors:** Rui Yang, Ying Guan, Jinwei Zhou, Bing Sun, Zhennan Wang, Hongjuan Chen, Zhaochun He, Aiqun Jia

**Affiliations:** ^1^School of Environmental and Biological Engineering, Nanjing University of Science and Technology, Nanjing, China; ^2^State Key Laboratory of Marine Resource Utilization in South China Sea, Key Laboratory of Tropical Biological Resources of Ministry of Education, Hainan University, Haikou, China; ^3^Inspection and Pattern Evaluation Department, Suzhou Institute of Measurement and Testing, Suzhou, China; ^4^State Key Laboratory of Pharmaceutical Biotechnology, Nanjing University, Nanjing, China

**Keywords:** *Camellia nitidissima* Chi flowers, phytochemicals, *Pseudomonas aeruginosa* PAO1, virulence factors, real-time RT-PCR, HPLC triple TOF MS/MS

## Abstract

*Camellia nitidissima* Chi, known as a medicinal and edible plant in China, exhibits multiple bioactivities, especially antibacterial activity. In this study, we investigated the inhibitory effects of the dichloromethane fraction (DF) of *C. nitidissima* Chi flowers on the pyocyanin production, swarming motility, and swimming motility of *Pseudomonas aeruginosa* PAO1, at sub-minimum inhibitory concentrations. Results showed that the DF had a remarkable inhibitory effect on pyocyanin production without influencing *P. aeruginosa* PAO1 growth, and concentration-dependent inhibitory effects on swarming and swimming motility. The half maximal inhibitory concentrations (IC_50_) were 0.158 ± 0.009, 0.139 ± 0.004, and 0.334 ± 0.049 mg/mL for pyocyanin production, swarming motility, and swimming motility, respectively. Real-time RT-PCR showed that the DF significantly down-regulated the expressions of *lasR* (*p* < 0.05) and *rhlR* (*p* < 0.01). In addition, gallic acid, catechin, ellagic acid, chlorogenic acid, quercetin, and kaempferol were identified in the DF by HPLC Triple TOF MS/MS analysis. All six identified compounds showed inhibitory effects on pyocyanin production, swarming motility, and swimming motility, though ellagic acid showed the strongest effects, with IC_50_ values of 0.067 ± 0.002, 0.024 ± 0.008, and 0.020 ± 0.003 mg/mL, respectively. Thus, the inhibitory effects on *P. aeruginosa* PAO1 virulence factors might be attributable to these six and/or other compounds in the DF of *C. nitidissima* Chi flowers. Consequently, the *C. nitidissima* Chi flower, especially the DF, might be a potential quorum sensing inhibitor of *P. aeruginosa* PAO1.

## Introduction

*Pseudomonas aeruginosa*, which thrives in soil and water, is an opportunistic pathogen of plants, animals, and humans (Parsek and Greenberg, 2000). It is one of the main factors responsible for nosocomial infections in humans, with a high incidence of infection occurring in immunocompromised patients, such as those who are intubated, have prosthetic devices, bear severe burns, or suffer from cystic fibrosis (Castillo-Juárez et al., 2015; Castillo-Juarez et al., 2017). Unfortunately, *P. aeruginosa* can cause infections that are difficult to treat due to its increasing resistance to antibiotics and the formation of biofilms on abiotic and biotic surfaces (Costerton, 2001; Drenkard, 2003; Schuster and Greenberg, 2006). The virulence factors of *P. aeruginosa*, such as pyocyanin, rhamnolipid, protease, elastase, and alginate, are complex multifactorial phenomena, which are influenced by the environment and quorum sensing (QS) (Castillo-Juarez et al., 2017). QS is a form of cell-to-cell communication, which monitors bacterial population density and several physiological processes (Schauder and Bassler, 2001; Sheng et al., 2015). Small signals called autoinducers are released and received in the QS system, and are most frequently N-acyl-homoserine lactones in Gram-negative bacteria or peptides in Gram-positive bacteria (Kalia, 2013). QS systems regulate multiple bacterial functions, such as virulence gene expression, swarming motility, and swimming motility (Kalia, 2013; Wang et al., 2013). *P. aeruginosa* has three QS systems, *las, rhl*, and *pqs* (Zhou et al., 2017). In the *las* system, the *lasI* gene encodes the signal synthase LasI, which produces the autoinducer N-3-oxo-dodecanoyl-homoserine lactone (3-oxo-C_12_-HSL), and the *lasR* gene encodes the signal receptor LasR, with the binding of LasI and LasR then activating other genes, including alkaline protease and elastase B (Gambello and Iglewski, 1991; Castillo-Juarez et al., 2017). In the *rhl* system, the *rhlI* gene encodes the enzyme RhlI, and the *rhlR* gene encodes the signal receptor RhlR, with the binding of RhlI and RhlR then producing and sensing the autoinducer N-butanoyl-homoserine lactone (C_4_-HSL) to regulate the expression of virulence genes (Ochsner et al., 1994; Castillo-Juarez et al., 2017). In the *pqs* system, the autoinducer 2-heptyl-3-hydroxy-4(1*H*)-quinolone is known as the *Pseudomonas* quinolone signal (PQS). The three systems are intertwined in the QS hierarchy. The LasR initiates the QS regulatory systems and partially activates the transcription of RhlR and other regulators of the *Pseudomonas* quinolone signal and integrated QS systems (Maisuria et al., 2016).

Recently, inhibition of the QS system has been considered as a novel strategy for the development of antipathogenic agents, especially for combating bacterial infections caused by antibiotic-resistant strains (Rasko and Sperandio, 2010). Quorum sensing inhibitors (QSIs) can reduce virulence factors but do not kill bacteria, thus avoiding the development of the resistance observed for antibiotics (Hentzer and Givskov, 2003). These inhibitors include non-functional A-HSL analogs, such as brominated furanones, which can bind to receptors of A-HSLs competitively (Hentzer et al., 2003), and synthetic non-functional homoserine lactones, which can interfere with binding between autoinducers and their receptors (Reverchon et al., 2002). In addition, the inactivation of A-HSL receptors via covalent modification can also inhibit QS (Castillo-Juarez et al., 2017). Recently, the development of QSIs from natural biological material, especially medicinal and edible plants such as garlic, pea seedlings, pomegranate extract, and *R. rugosa* tea extract, has gained increasing attention (Puupponen-Pimia et al., 2005; Choo et al., 2006; Truchado et al., 2012; Zhang et al., 2014). As an important and widely distributed constituent of plants, phenolic compounds possess many important physiological functions and have recently been reported to reduce QS-controlled phenotypes in bacteria (Zhang et al., 2014; Ouyang et al., 2016; Skogman et al., 2016). In addition, bacterial motility plays an important role in the pathogenidty. *P. aeruginosa* has at least three types of motilities, including swarming, swimming, and twitching (Wang et al., 2014). So it is also a strategy for the development of antipathogenic agents to investigate the motility inhibitors. And it is reported that many compounds and extracts, such as zingerone (Kumar et al., 2015), 2,5-piperazinedione (Musthafa et al., 2012), macrolides (Kawamurasato et al., 2000), atorvastatin, rosuvastatin (Dhaliwal, 2015), and *Agaricus Blazei* hot water extract (Sokovic et al., 2014), are regarded as motility inhibitors to inhibit the bacterial motility.

*Camellia nitidissima* Chi, a popular medicinal and edible plant in China, is distributed in a narrow region of Southern China and Northern Vietnam. Its flowers, leaves, and seed oils show multiple bioactivities; for example, *C. nitidissima* flowers have been shown to inhibit the growth of the Eca109 human esophageal squamous cell carcinoma cell line (Dai et al., 2016), whereas its leaves reportedly inhibit the formation of advanced glycation end-products (Wang et al., 2016), and its seeds have been found to exhibit cytotoxicity against human lymphoma cells, as well as cervical and prostate cancer cells (Han et al., 2009). In addition, *C. nitidissima* Chi has shown antimicrobial effects against *Staphylococcus albus, Beta streptococcus, Corynebacterium diphtheriae*, and *P. aeruginosa* (Chen et al., 2009). However, the effects of *C. nitidissima* Chi flowers on QS-controlled phenotypes in bacteria and whether the flowers are a potential QSI remain unknown. Accordingly, we investigated the inhibitory effects of *C. nitidissima* Chi flower fractions on *P. aeruginosa* PAO1 virulence factors.

## Materials and methods

### Bacterial strains and materials

*P. aeruginosa* PAO1 was kindly donated by Prof. Q.H. Gong from the Ocean University of China in Qingdao, and was incubated in nutrient broth (NB) at 37°C unless otherwise specified. The *C. nitidissima* Chi flowers were collected in July 2016 from a cultivated farm in Fangchenggang, Guangxi, China, and stored at 4°C. A *C. nitidissima* Chi flower voucher specimen (JHCH-001) was deposited in our lab. Gallic acid, catechin, ellagic acid, chlorogenic acid, quercetin, and kaempferol were purchased from Sangon Biotech Co., Ltd. (Shanghai, China) and dissolved in pure dimethyl sulfoxide (DMSO), the concentrations were 30, 30, 10, 30, 20, and 10 mg/mL, respectively. All other reagents in this study were of analytical grade.

### Phytochemical extraction preparation

Phytochemical extraction procedures followed those of previous research (Wang et al., 2016), with some modifications. The *C. nitidissima* Chi flowers (500 g) were sun dried, then refluxed with 95% ethanol and evaporated in a rotary evaporator at 45°C to obtain the ethanolic extract (EE, 165 g). The EE was then suspended in water and extracted with dichloromethane, ethyl acetate, and n-butanol to yield the dichloromethane fraction (DF, 5.72 g), ethyl acetate fraction (EAF, 29.3 g), and n-butanol fraction (BF, 61.7 g), with the residue evaporated at 45°C to obtain the water fraction (WF, 33 g).

### Minimum inhibitory concentrations (MICs)

The MIC values of the five fractions and the six identified compounds were determined as per previously published methods (Zhou et al., 2017), with some modifications. Briefly, overnight culture of *P. aeruginosa* PAO1 (1%, v/v) were added to Mueller-Hinton Broth supplemented with the samples at concentration gradients (two-fold dilution, 0.05–5.0 mg/mL) in 96-well microtiter plates, then incubated at 37°C and 150 rpm for 15 h. The MIC was the lowest concentration of the samples with visible inhibition of cell growth. The MICs of the five fractions and six identified compounds against *P. aeruginosa* PAO1 are recorded in Table 1. All further experiments in this study were conducted at sub-MICs.

**Table 1:** Minimum inhibitory concentrations (MICs) of the five fractions and six compounds identified from *Camellia nitidissima* Chi flowers against *Pseudomonas aeruginosa* PAO1.

**Fractions**	**MIC values (mg/mL)**	**Identified compounds**	**MIC values (mg/mL)**
Ethanolic extract	2.50	Gallic acid	>2.50
Dichloromethane fraction	2.50	Catechin	>2.50
Ethyl acetate fraction	5.00	Ellagic acid	0.15
n-butanol fraction	1.25	Chlorogenic acid	0.35
Water fraction	1.25	Quercetin	0.25
		Kaempferol	0.15

### Determination of pyocyanin production

Pyocyanin production was determined as per previously published methods (O'Loughlin et al., 2013), with some modifications. The *P. aeruginosa* PAO1 culture was incubated at 37°C overnight, with 20 μL of the overnight culture then added to 2 mL of fresh medium (2% peptone, 0.14% MgCl_2_, 1% K_2_SO_4_, and 1% glycerinum, pH 7.4) supplemented with the five fractions and six identified compounds with shaking at 37°C and 150 rpm for 17 h. The concentrations of the five fractions were 0.0625, 0.125, 0.25, 0.5, and 0.75 mg/mL, the concentrations of gallic acid, catechin, and chlorogenic acid were 0.0375, 0.075, 0.15, and 0.3 mg/mL, the concentrations of quercetin were 0.025, 0.05, 0.1, and 0.2 mg/mL, and the concentrations of ellagic acid and kaempferol were 0.0125, 0.025, 0.05, and 0.1 mg/mL. DMSO was used as the control (0.75%, v/v). Cells were separated from culture fluids via centrifugation at 12,000 rpm for 15 min at 4°C. The cell-free culture fluids were then analyzed for pyocyanin production at 695 nm using a spectrophotometer (BioTek, Vermont, USA).

### Bacterial growth measurement

The effect of the DF and six identified compounds on the growth of *P. aeruginosa* PAO1 were measured following previous methods (Sheng et al., 2015), with some modifications. In brief, overnight culture of *P. aeruginosa* PAO1 (1%, v/v) were added to NB supplemented with the DF at concentration gradients (0, 0.0625, 0.125, 0.25, 0.5, and 0.75 mg/mL) in Erlenmeyer flasks, then incubated at 37°C and 150 rpm. And the concentrations of gallic acid, catechin, ellagic acid, chlorogenic acid, quercetin, and kaempferol were 300, 300, 100, 300, 200, and 100 μg/mL, respectively. DMSO was used as the control (1%, v/v). The OD_620_ values of the culture were measured every 2 h for 24 h by a microplate reader (Biotek Elx800, USA). The growth of *P. aeruginosa* PAO1 was evaluated by plotting the values of OD_620_ against time.

### Swarming assay

The swarming assay was conducted as per prior published methods (Sheng et al., 2015), with some modifications. Briefly, the DF and six identified compounds were added to molten swarming agar (pH 7.2), which consisted of NB (0.8%), glucose (0.5%), and bacto-agar (0.5%). The concentrations of the five fractions were 0.0625, 0.125, 0.25, 0.5, and 0.75 mg/mL, the concentrations of gallic acid, catechin, and chlorogenic acid were 0.0375, 0.075, 0.15, and 0.3 mg/mL, the concentrations of quercetin were 0.025, 0.05, 0.1, and 0.2 mg/mL, and the concentrations of ellagic acid and kaempferol were 0.0125, 0.025, 0.05, and 0.1 mg/mL. The culture was then dispensed onto Petri dishes after gentle mixing. Once the culture was solidified, 2 μL of overnight *P. aeruginosa* PAO1 culture was inoculated in the center of the agar and then incubated at 37°C for 24 h. We used DMSO as the control (1%, v/v). Anti-QS properties were identified by the reduction in swarming motility.

### Swimming assay

The swimming assay was conducted according to previous research (Luo et al., 2016), with some modifications. The procedures were the same as those of the swarming assay, except for the swimming agar (pH 7.2), which consisted of peptone (1.0%), sodium chloride (0.5%), and bacto-agar (0.3%).

### Real-time RT-PCR

The real-time RT-PCR procedures were conducted following previous studies (Yang et al., 2012; Sheng et al., 2015), with some modifications. Overnight *P. aeruginosa* PAO1 culture was diluted (1:1,000) into fresh NB, with the DF added to a final concentration of 0.75 mg/mL. Cells were collected after incubation at 37°C for 16 h with agitation. Total RNA was extracted using an RNeasy Mini Kit (Qiagen, Germany) according to the manufacturer's protocols. The RNA was then reverse transcribed into complementary DNA (cDNA) using a HiScript® Q RT SuperMix for qPCR (+gDNA wiper) (Vazyme, China) according to the manufacturer's instructions. The real-time RT-PCR was performed in a 20 μL volume using an AceQTM qPCR SYBR® Green Master Mix (Vazyme, China) as recommended by the manufacturer. Primers, used to amplify the QS circuit genes *lasI, lasR, rhlI*, and *rhlR* and reference gene *rpsL*, are shown in Table 2. The reaction was performed using the Applied Biosystems 7300 RT-PCR System (USA) and involved incubation at 95°C for 5 min, 40 cycles at 95°C for 15 s, 58°C for 30 s, and 72°C for 30 s. The expressions of the target genes were normalized to the expression of the reference gene *rpsL*.

**Table 2:** Primers used for quorum sensing circuit genes *lasI, lasR, rhlI*, and *rhlR*, and reference gene, *rpsL*.

**Gene**	**Type**	**Primer sequence**	**Amplicon size (bp)**
*lasI*	F	GGCTGGGACGTTAGTGTCAT	104
	R	AAAACCTGGGCTTCAGGAGT	
*lasR*	F	ACGCTCAAGTGGAAAATTGG	111
	R	TCGTAGTCCTGGCTGTCCTT	
*rhlI*	F	AAGGACGTCTTCGCCTACCT	130
	R	GCAGGCTGGACCAGAATATC	
*rhlR*	F	CATCCGATGCTGATGTCCAACC	101
	R	ATGATGGCGATTTCCCCGGAAC	
*rpsL*	F	GCAACTATCAACCAGCTGGTG	231
	R	GCTGTGCTCTTGCAGGTTGTG	

### Dichloromethane fraction assay by HPLC triple TOF MS/MS

We used HPLC Triple TOF MS/MS for the DF assay, as per previously published methods (Wang et al., 2016), with some modifications. The DF was analyzed on a Shimadzu HPLC equipped with a diode array detector, and a Welch Ultimate XB-C18 column (100 × 2.1 mm i.d., 3 μm; Welch Materials, Inc., Shanghai, China). Mobile phase A was 0.1% formic acid of water and mobile phase B was 0.1% formic acid of methanol, and the linear gradient was 0–1 min, 5–5% B; 1–30 min, 5–70% B; 30–35 min, 70–90% B; 35–40 min, 90–90% B; 40–40.1 min, 90–5% B; 40.1–45 min, 5–5% B. The flow rate was 0.4 mL/min and the injection volume was 10 μL. The Triple TOF 4600 system (AB SCIEX, CA) with electrospray ionization was operated at negative mode. The following parameter settings were used: ion spray voltage, 4.5 kV; ion source heater, 550°C; curtain gas, 25 psi; ion source gas 1, 55 psi; and ion source gas 2, 55 psi. Mass spectra were scanned from m/z 100 to 1,500. The collision energy was swept from 30 to 60 eV for MS/MS analysis.

### Statistical analyses

All experiments were conducted independently with at least three replicates, and results were expressed as means ± standard deviation or average. Interpolation from linear regression analysis was used to obtain the half maximal inhibitory concentrations (IC_50_). Unpaired or two-tailed paired *t-*tests were used to evaluate the significance of differences between two groups. One-way analysis of variance (ANOVA) and Duncan's multiple range tests were performed using SPSS version 17.0 (SPSS Inc., Chicago, IL, USA) software. Statistical significance was determined at *p* < 0.05.

## Results and discussion

### *Camellia nitidissima* chi flowers inhibit pyocyanin production

**Figure 1:**
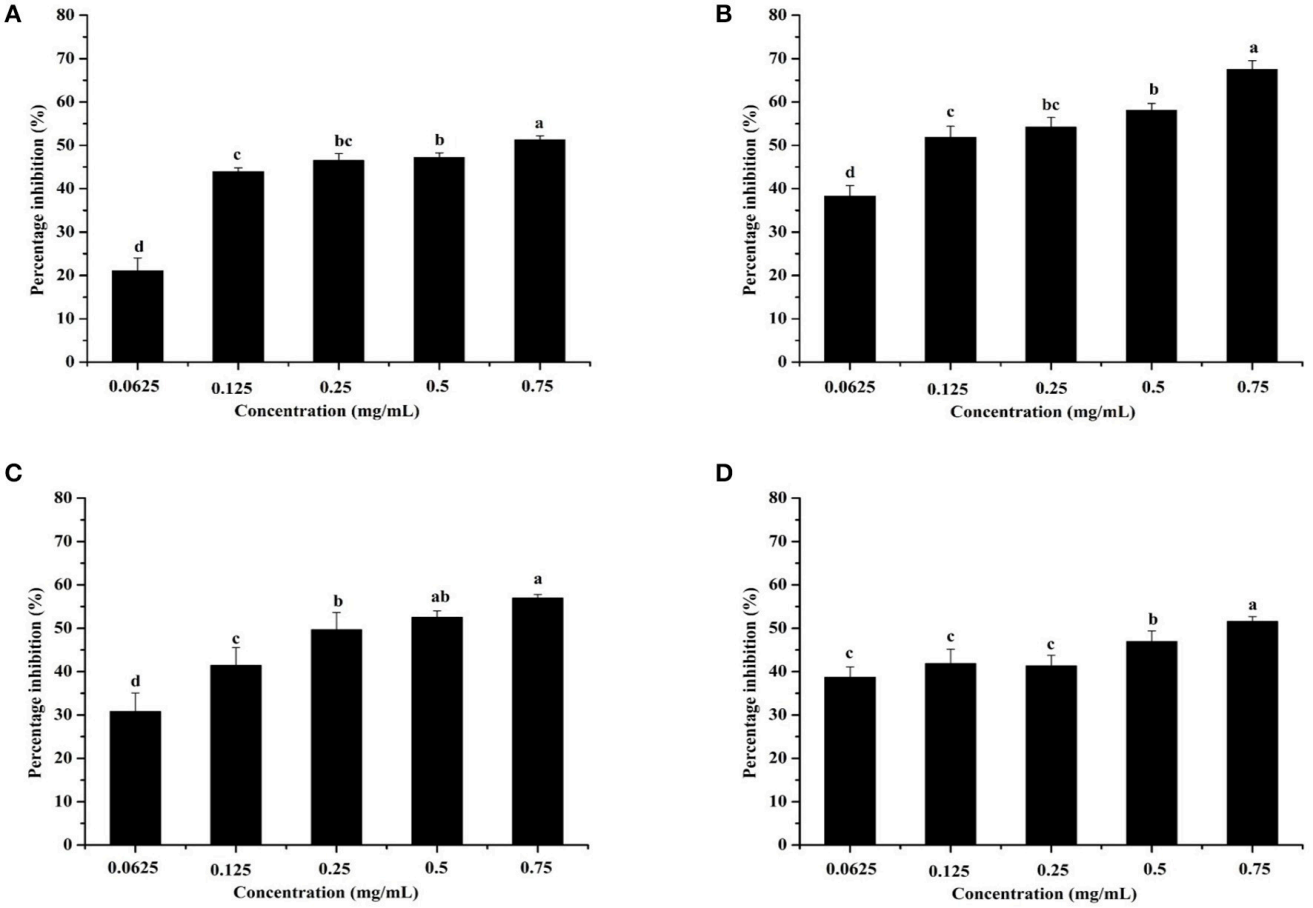
Effects of the *Camellia nitidissima* Chi flower fractions on pyocyanin production. Ethanolic extract **(A)**, Dichloromethane fraction **(B)**, Ethyl acetate fraction **(C)**, and N-butanol fraction **(D)**. Means with different small letters (a–d) are significantly different (*p* < 0.05).

**Figure 2:**
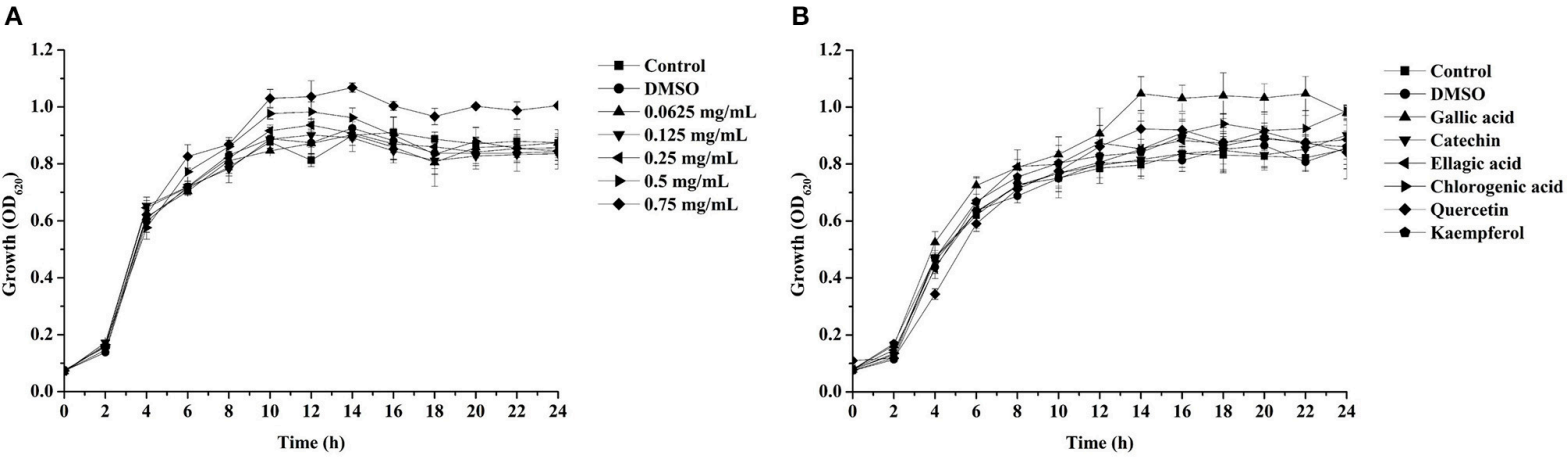
Effects of the dichloromethane fraction from the *Camellia nitidissima* Chi flower **(A)** and gallic acid (300 μg/mL), catechin (300 μg/mL), ellagic acid (100 μg/mL), chlorogenic acid (300 μg/mL), quercetin (200 μg/mL), and kaempferol (100 μg/mL) **(B)** on *Pseudomonas aeruginosa* PAO1 growth.

Pyocyanin is a vital QS-regulated virulence factor of *P. aeruginosa* PAO1. The inhibitory effects of the *C. nitidissima* Chi flower fractions on pyocyanin production are shown in Figure 1. The WF showed no inhibition activity, whereas the other four fractions showed remarkable concentration-dependent inhibitory effects on pyocyanin production. Of note, the DF had the highest inhibiting effect on pyocyanin production. At a concentration of 0.75 mg/mL, the percentage of inhibition was 67.511 ± 2.035 for the DF, and 51.265 ± 0.949, 56.962 ± 0.837, and 51.582 ± 1.096, respectively, for the EE, EAF, and BF. In addition, the IC_50_ value of the DF on pyocyanin production (0.158 ± 0.009 mg/mL) was significantly (*p* < 0.05) lower than that of the other three fractions (Table 3). As seen in Figure 2A, the DF had no influence on the growth of *P. aeruginosa* PAO1 at the serial concentrations of 0.0625–0.75 mg/mL. We selected the DF for all further experiments in this study due to its highest activity. As a famous tea, the *C. nitidissima* Chi flowers are known to have a high content of tea phenolic compounds (Peng et al., 2012). Phenolic compounds, such as ellagic acid, quercetin, and catechin, are able to inhibit pyocyanin production (Vandeputte et al., 2010; Sarabhai et al., 2013; Ouyang et al., 2016). Therefore, the inhibitory effects of *C. nitidissima* Chi flowers on pyocyanin production observed here might be via their phenolic compounds. In addition, pyocyanin is encoded by virulence genes, which are regulated by the RhlRI QS system in *P. aeruginosa* PAO1 (Castillo-Juarez et al., 2017). Thus, some compounds in the *C. nitidissima* Chi flowers, especially in the DF, might influence the expressions of *rhlI* and/or *rhlR* to inhibit the production of pyocyanin in *P. aeruginosa* PAO1.

**Table 3:** Half maximal inhibitory concentrations (IC_50_) of four fractions and six compounds identified from *Camellia nitidissima* Chi flowers on pyocyanin production of *Pseudomonas aeruginosa* PAO1.

**Samples**	**IC_50_ (mg/mL)**
Ethanolic extract	0.520 ± 0.041b
Dichloromethane fraction	0.158 ± 0.009e
Ethyl acetate fraction	0.347 ± 0.058c
n-butanol fraction	0.672 ± 0.015a
Gallic acid	0.212 ± 0.005d
Catechin	0.258 ± 0.023d
Ellagic acid	0.067 ± 0.002f
Chlorogenic acid	nd
Quercetin	0.147 ± 0.002e
Kaempferol	nd

*nd, Not detected*.

*IC_50_ values were obtained by interpolation from linear regression analysis. Values are presented as mean ± SD (n = 3), and means in the same column with different lower case letters (a–f) are significantly different (p < 0.05)*.

### Dichloromethane fraction inhibits *P. aeruginosa* PAO1 swarming motility

**Figure 3:**
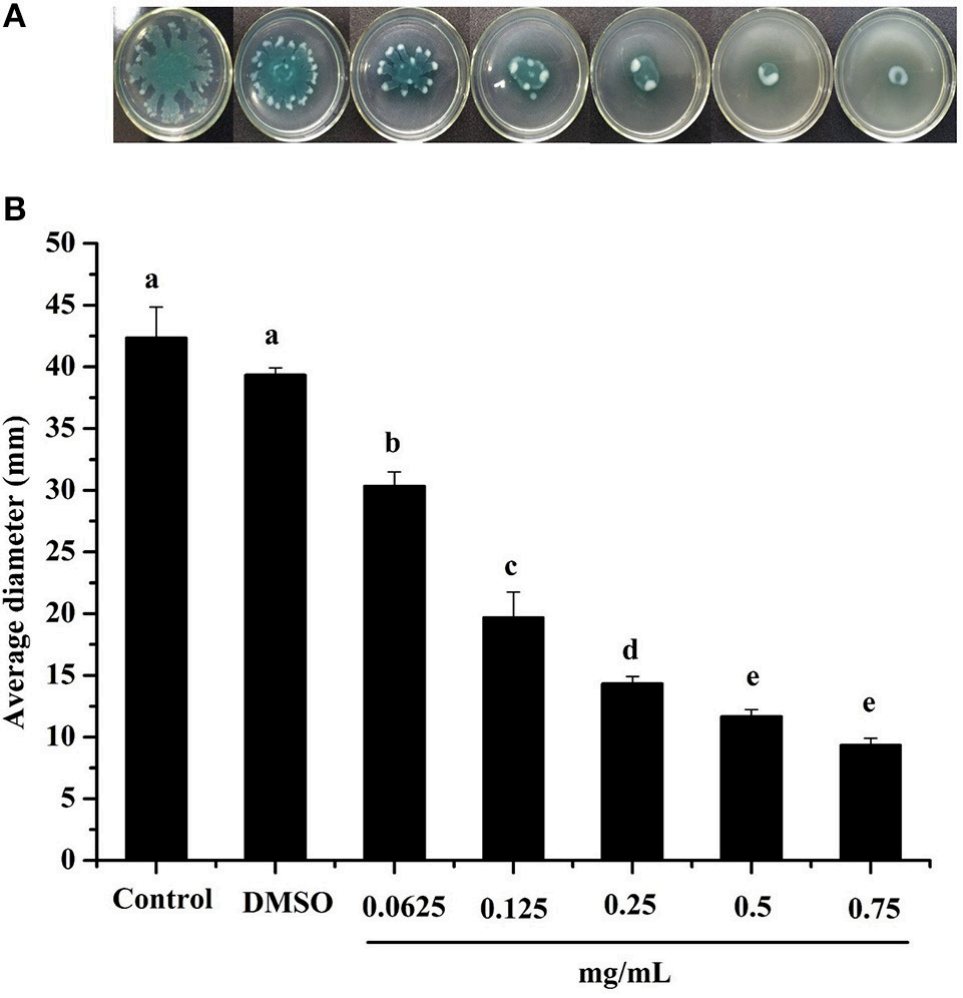
Swarming inhibition assay. Swarming agar inoculated with the dichloromethane fraction of the *Camellia nitidissima* Chi flowers **(A)**, and average diameter of the bacterial colony tendrils **(B)**. Means with different small letters (a–e) are significantly different (*p* < 0.05).

Swarming motility is a type of virulence factor in *P. aeruginosa* PAO1, and is defined by rapid and coordinated translocation of a bacterial population across a semi-solid surface (Hayouni et al., 2008). As seen in Figure 3, the DF significantly inhibited the swarming motility of *P. aeruginosa* PAO1 in a concentration-dependent manner. The tendrils of the *P. aeruginosa* PAO1 bacterial colony decreased with increasing DF concentration, and the IC_50_ value of the DF on swarming motility was 0.139 ± 0.004 mg/mL (Table 4). At 0.75 mg/mL, there were no defined tendrils observed, and the average swarming diameter was 9.333 ± 0.577 mm. The significant inhibition effect on swarming motility was also observed at the relatively low concentration of 0.0625 mg/mL, with an average swarming diameter of 30.333 ± 1.155 mm, which was significantly lower (*p* < 0.05) than that of the control (42.333 ± 2.517 mm). Swarming motility is a phenotype controlled by the QS system (Kohler et al., 2000). Therefore, our results strongly indicate that the DF had the ability to interfere with the QS system of *P. aeruginosa* PAO1.

**Table 4:** Half maximal inhibitory concentrations (IC_50_) of the dichloromethane fraction and six compounds identified from *Camellia nitidissima* Chi flowers on the swarming motility and swimming motility of *Pseudomonas aeruginosa* PAO1.

**Samples**	**IC**_**50**_ **(mg/mL)**	
	**Swarming motility**	**Swimming motility**
Dichloromethane fraction	0.139 ± 0.004b	0.334 ± 0.049a
Gallic acid	0.217 ± 0.018a	nd
Catechin	0.051 ± 0.006d	0.221 ± 0.009b
Ellagic acid	0.024 ± 0.008e	0.020 ± 0.003d
Chlorogenic acid	0.116 ± 0.014c	0.218 ± 0.006b
Quercetin	nd	0.164 ± 0.019c
Kaempferol	0.037 ± 0.002de	nd

*nd, Not detected*.

*IC_50_ values were obtained by interpolation from linear regression analysis. Values are presented as mean ± SD (n = 3), and means in the same column with different lower case letters (a–e) are significantly different (p < 0.05)*.

### Dichloromethane fraction inhibits *P. aeruginosa* PAO1 swimming motility

**Figure 4:**
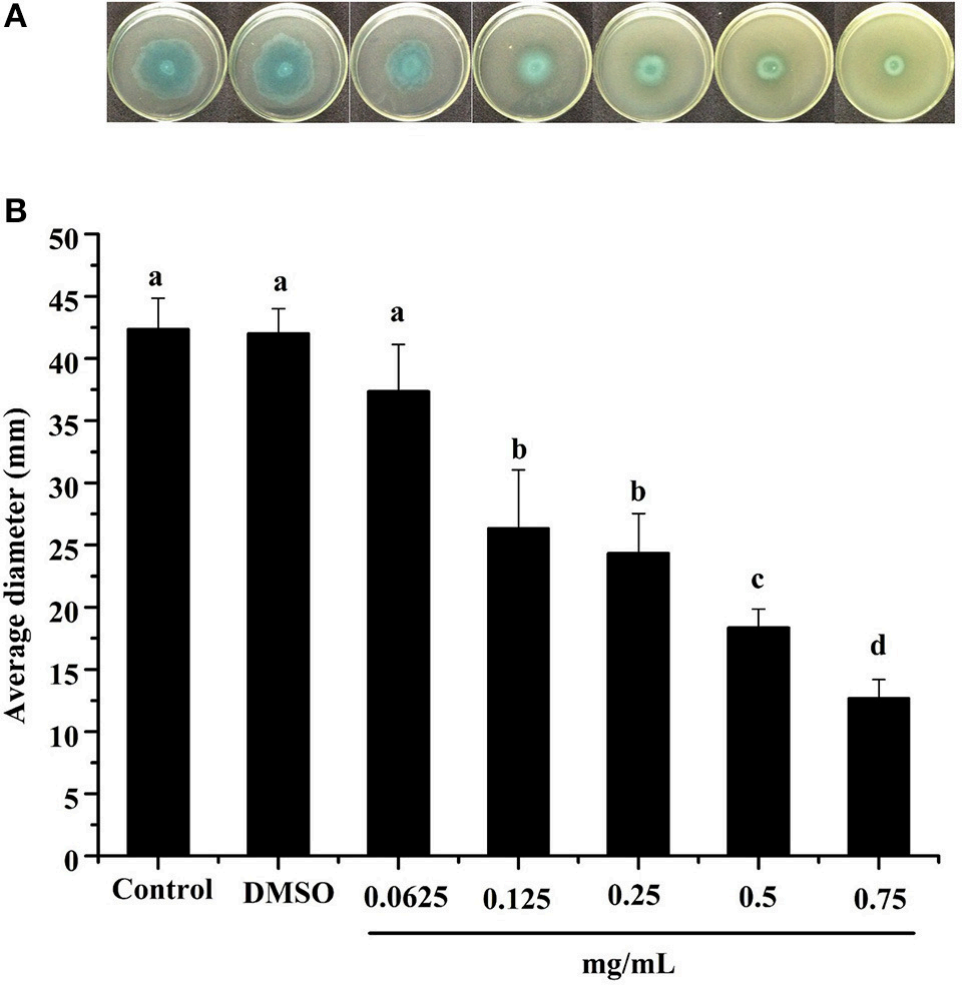
Swimming inhibition assay. Swimming agar inoculated with the dichloromethane fraction of the *Camellia nitidissima* Chi flowers **(A)**, and average diameter of the bacterial colony **(B)**. Means with different small letters (a–e) are significantly different (*p* < 0.05).

Swimming is another major form of *P. aeruginosa* PAO1 motility, in which bacteria swim in aqueous environments via the flagellum (Rashid and Kornberg, 2000). As shown in Figure 4, the DF inhibited *P. aeruginosa* PAO1 swimming motility in a concentration-dependent manner. The average diameters of the bacterial colony significantly (*p* < 0.05) decreased with increasing DF concentration, and were 37.33 and 12.67 mm at concentrations of 0.0625 and 0.75 mg/mL, respectively, compared with 42.33 mm for the control. The IC_50_ value of the DF on swimming motility was 0.334 ± 0.049 mg/mL (Table 4). Similar to swarming motility, swimming motility is regulated by the QS system in *P. aeruginosa* PAO1 (Williams and Camara, 2009; Kumar et al., 2015) and is crucial for its pathogenesis, playing an important role in the expression of full virulence and colonization. Thus, the DF might inhibit swimming motility by interfering with QS, thereby contributing to the reduced expression of *P. aeruginosa* PAO1 virulence factors.

### Dichloromethane fraction effects the expressions of *lasI, lasR, rhlI*, and *rhlR*

**Figure 5:**
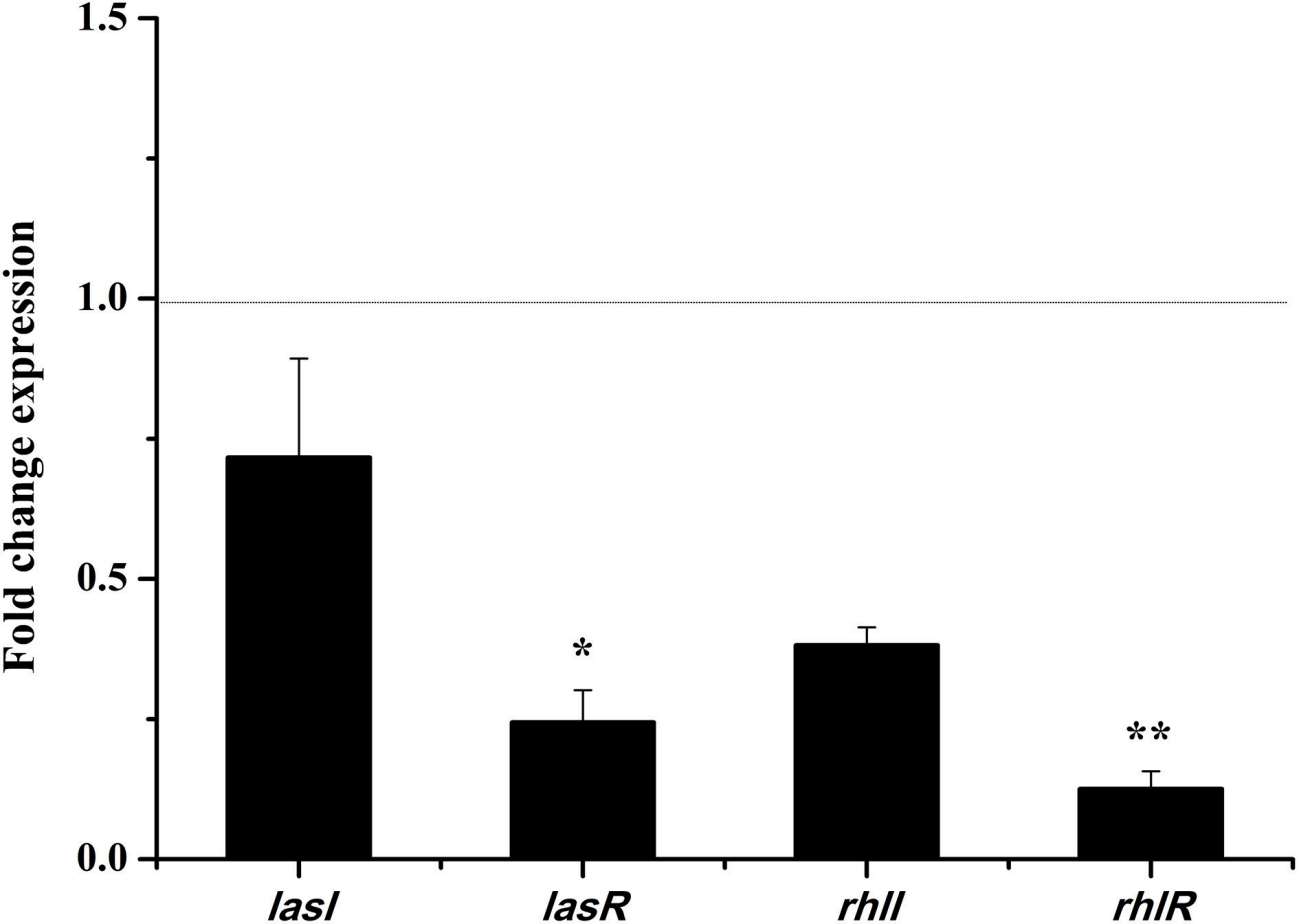
Effects of the dichloromethane fraction (0.75 mg/mL) of the *Camellia nitidissima* Chi flowers on gene expressions of QS regulatory circuits in *Pseudomonas aeruginosa* PAO1. Average relative amounts of tested genes were normalized to the average relative amount of the *rpsL* reference gene. ^*^p < 0.05; ^**^p < 0.01.

In the *las* and *rhl* systems, the virulence factors of *P. aeruginosa* PAO1 are mainly encoded by QS-regulated genes *lasI, lasR, rhlI*, and *rhlR* (Castillo-Juarez et al., 2017). We investigated whether the DF could influence the expressions of QS-regulated genes to reduce *P. aeruginosa* PAO1 virulence factors. As shown in Figure 5, the DF (at 0.75 mg/mL) down-regulated the expression of all tested genes. Average relative amounts of the tested genes were normalized to the average relative amount of the *rpsL* reference gene, with *lasR* (*p* < 0.05) and *rhlR* (*p* < 0.01) found to be significantly decreased. In the *las* system, the *lasR* gene encodes the signal receptor LasR, and binds 3-oxo-C12-HSL to activate certain target gene transcriptions (Pearson et al., 1994; Schuster and Greenberg, 2006). In the *rhl* system, the *rhlR* gene encodes the signal receptor RhlR, and induces gene expression when complexed with C4-HSL (Pearson et al., 1995; Schuster and Greenberg, 2006). It has been reported that RhlR antagonists can strongly inhibit pyocyanin production (O'Loughlin et al., 2013), and that LasR and RhlR interacting with and activated by 3-oxo-C12-HSL and C4-HSL, respectively, can trigger the production of pyocyanin and other virulence factors (Vandeputte et al., 2011). Thus, in this study, the significantly decreased expressions of *lasR* (*p* < 0.05) and *rhlR* (*p* < 0.01) resulted in the decrease in pyocyanin production and swarming and swimming motility. Our results indicate that the DF could reduce *P. aeruginosa* PAO1 virulence factors via regulation of the QS system.

### Dichloromethane fraction assay by HPLC triple TOF MS/MS

**Figure 6:**
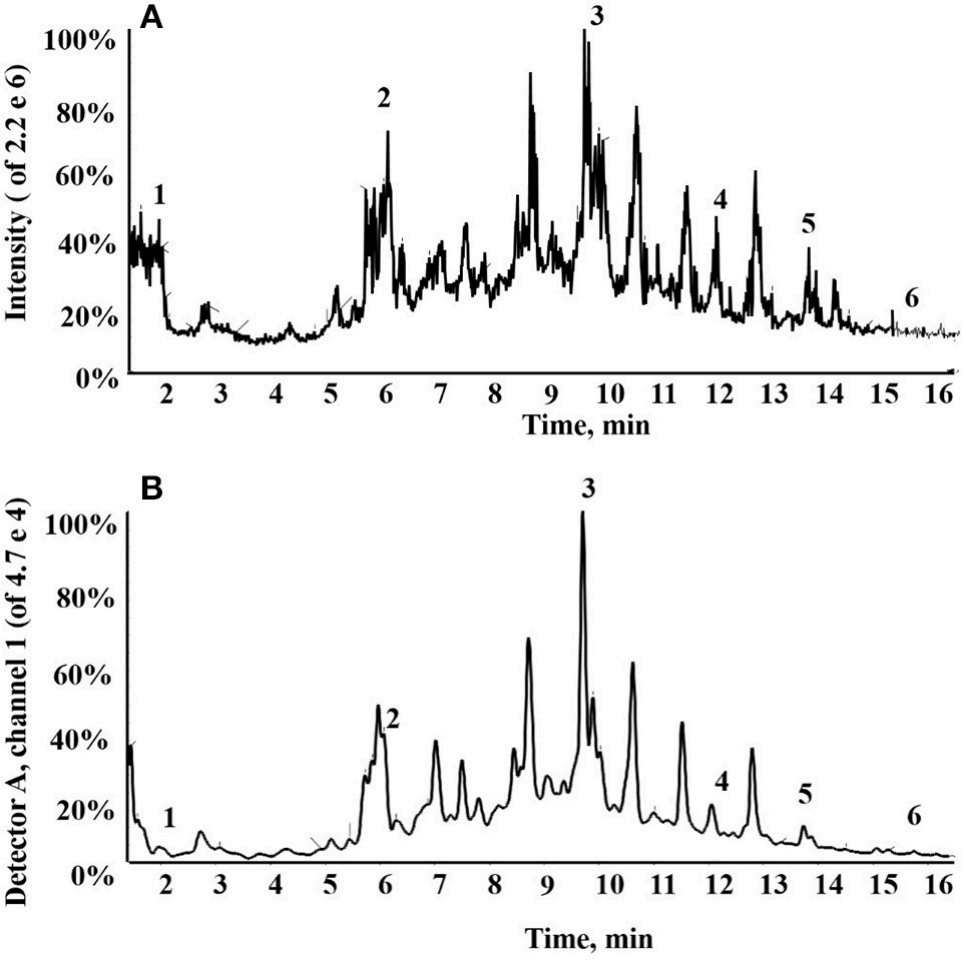
Total ion chromatogram of the dichloromethane fraction of the *Camellia nitidissima* Chi flowers **(A)**; HPLC chromatogram of the dichloromethane fraction by 360 nm detection **(B)**.

**Figure 7:**
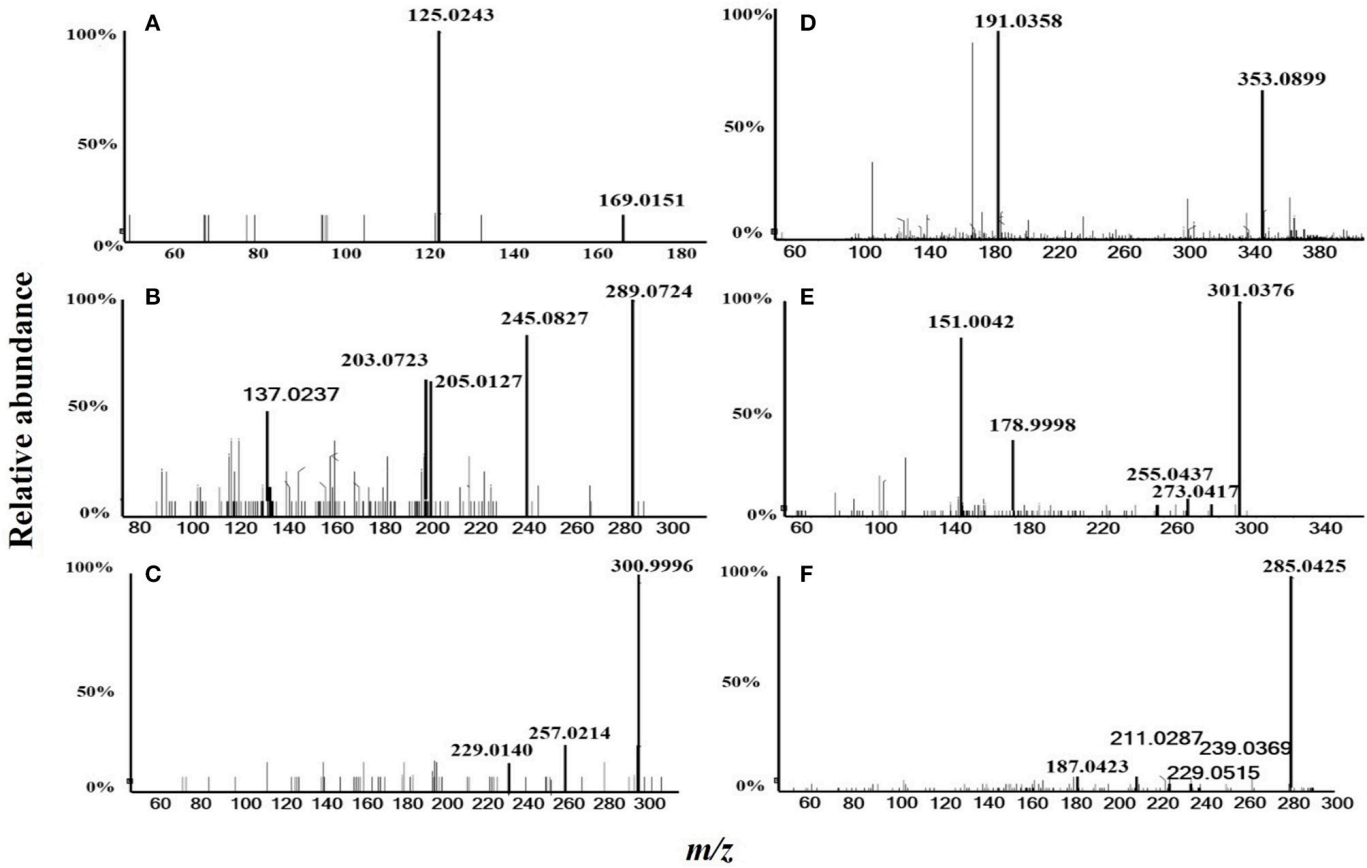
MS/MS spectrometric analysis of the six compounds identified in the dichloromethane fraction of the *Camellia nitidissima* Chi flowers by HPLC Triple TOF MS/MS. Gallic acid **(A)**, Catechin **(B)**, Ellagic acid **(C)**, Chlorogenic acid **(D)**, Quercetin **(E)**, and Kaempferol **(F)**.

In total, six compounds in the DF of *C. nitidissima* Chi flowers were identified (Table 5) by HPLC Triple TOF MS/MS analysis. The extract ion chromatogram at m/z 169.0146 showed a peak at R_t_ 2.09 min (Figure 6). The peak displayed a fragment at m/z 125 (Figure 7A) corresponding to the loss of one CO_2_ fragment, and was identified as gallic acid (Dou et al., 2007). At m/z 289.0726, the chromatogram showed a peak at R_t_ 6.14 min (Figure 6). The peak displayed fragments at m/z 245, 205, 203, and 137 (Figure 7B) corresponding to the loss of CO_2_, C_4_H_4_O_2_, C_4_H_6_O_2_, and C_8_H_8_O_3_ fragments, respectively, and was identified as catechin (Gottumukkala et al., 2014). At m/z 300.99931, the chromatogram showed a peak at R_t_ 9.76 min (Figure 6). The peak displayed fragments at m/z 257 and 229 (Figure 7C), and was identified as ellagic acid based on the mass spectra (Mullen et al., 2003). At m/z 353.08916, the chromatogram showed a peak at R_t_ 12.34 min (Figure 6). The peak displayed a fragment at m/z 191 (Figure 7D), and was identified as chlorogenic acid (Fang et al., 2002). At m/z 301.0366, the chromatogram showed a peak at R_t_ 13.86 min (Figure 6), with fragments at m/z 273, 255, 179, and 151 (Figure 7E) corresponding to the loss of CO, CH_2_O_2_, C_7_H_6_O_2_, and C_8_H_6_O_3_ fragments, respectively. The compound was identified as quercetin (McNab et al., 2009). At m/z 285.0405, the chromatogram showed a peak at R_t_ 15.86 min (Figure 6), with fragments at m/z 239, 229, 211, and 187 (Figure 7F), and was identified as kaempferol (McNab et al., 2009).

**Table 5:** Mass spectrometric data of the six compounds identified in the dichloromethane fraction of *Camellia nitidissima* Chi flowers by HPLC Triple TOF MS/MS.

**Peak**	**R_t_/min**	**Molecular formula**	**Tentative identification**	**Calculated [M-H]^−^**	**Measured [M-H]^−^**	**Error/ppm**	**MS/MS**
1	2.09	C_7_H_6_O_5_	Gallic acid	169.01425	169.01456	1.9	125
2	6.14	C_15_H_14_O_6_	Catechin	289.07176	289.0726	2.9	245, 205, 203, 137
3	9.76	C_14_H_6_O_8_	Ellagic acid	300.99899	300.99931	1.1	257, 229
4	12.34	C_16_H_18_O_9_	Chlorogenic acid	353.08781	353.08916	3.8	191
5	13.86	C_15_H_10_O_7_	Quercetin	301.03538	301.03661	4.1	273, 255, 179, 151
6	15.86	C_15_H_10_O_6_	Kaempferol	285.04046	285.04049	0.1	239, 229, 211, 187

### Effects of the six identified compounds on reducing *P. aeruginosa* PAO1 pyocyanin production and motility

**Figure 8:**
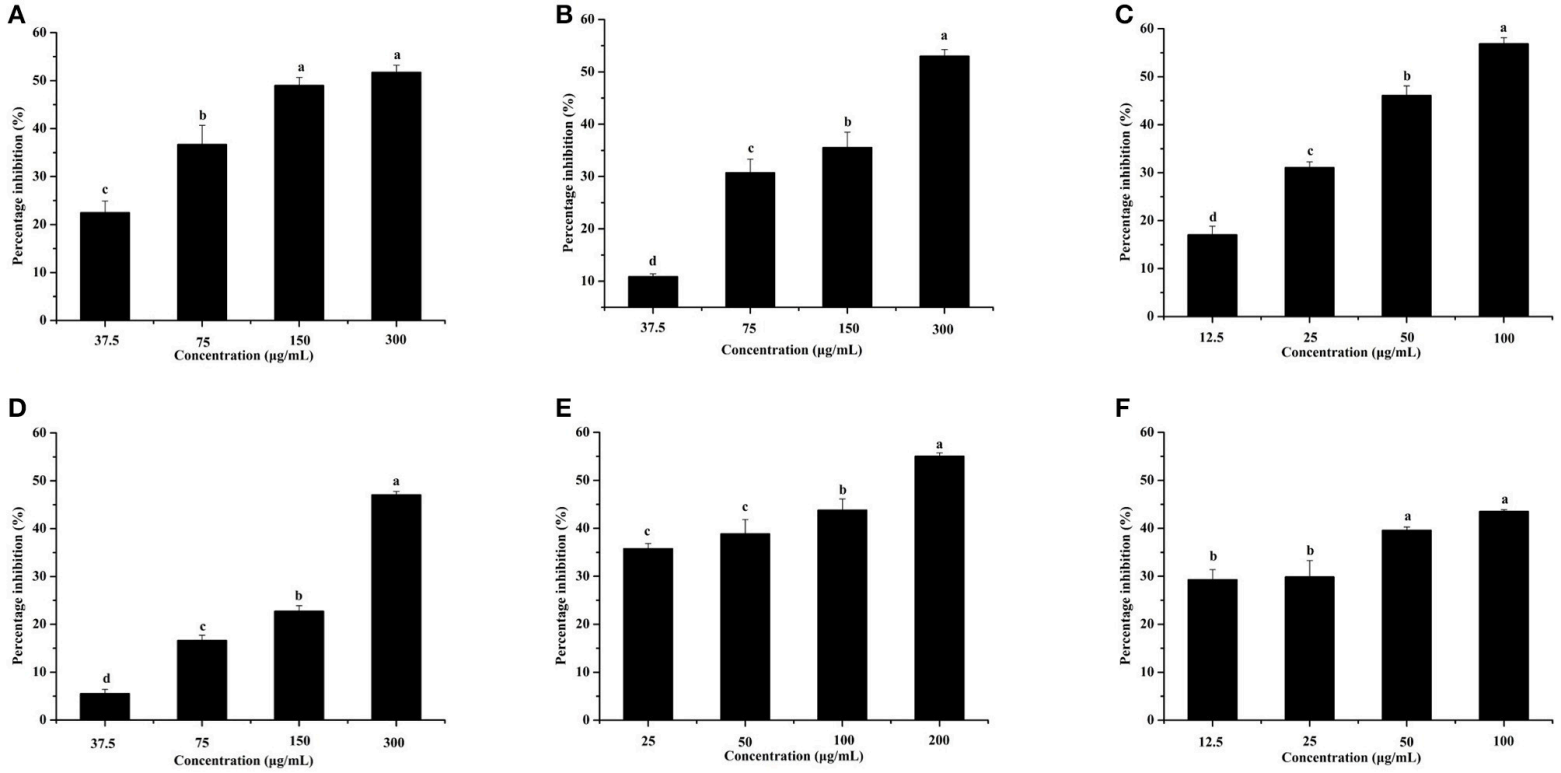
Effects of the six identified compounds on pyocyanin production. Gallic acid **(A)**, Catechin **(B)**, Ellagic acid **(C)**, Chlorogenic acid **(D)**, Quercetin **(E)**, and Kaempferol **(F)**. Means with different small letters (a–d) are significantly different (*p* < 0.05).

The effects of the six identified compounds on reducing *P. aeruginosa* PAO1 pyocyanin production are shown in Figure 8. Obviously, all six identified compounds had the ability to reduce pyocyanin production without the effects on the growth (Figure 2B), and except for kaempferol, the effects of the compounds were in a concentration-dependent manner. Among the six identified compounds, ellagic acid showed the strongest effect on reducing pyocyanin production, with a percentage inhibition of 52.3% at 0.10 mg/mL. The IC_50_ of ellagic acid on pyocyanin production was 0.067 ± 0.002 mg/mL (Table 3), which was significantly (*p* < 0.05) lower than that of the other five compounds. The IC_50_ of quercetin on pyocyanin production was 0.147 ± 0.002 mg/mL, which was significantly (*p* < 0.05) lower than that of gallic acid and catechin (0.212 ± 0.005 and 0.258 ± 0.023 mg/mL, respectively). The IC_50_ values of chlorogenic acid and kaempferol on pyocyanin production were not detected. These results suggest that the four compounds, especially ellagic acid, in the DF played important roles in the inhibition of *P. aeruginosa* PAO1 pyocyanin production.

**Figure 9:**
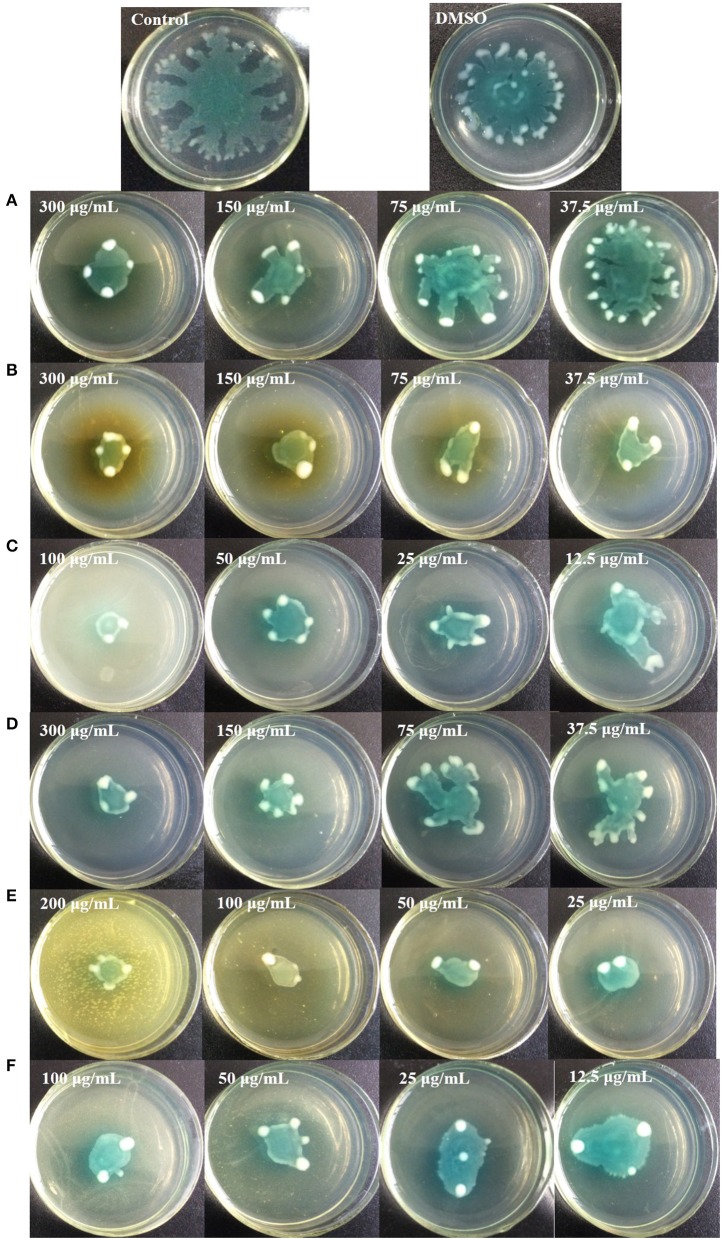
Swarming inhibition assays of the six identified compounds. Gallic acid **(A)**, Catechin **(B)**, Ellagic acid **(C)**, Chlorogenic acid **(D)**, Quercetin **(E)**, and Kaempferol **(F)**.

**Figure 10:**
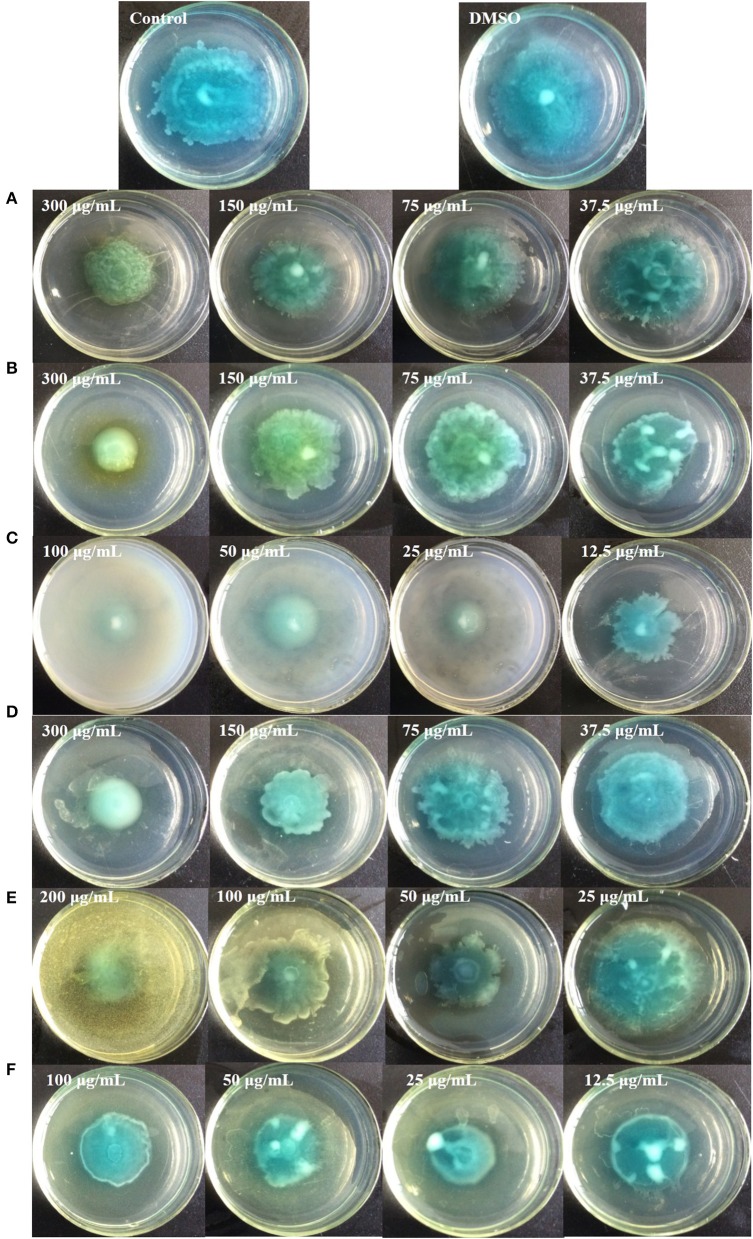
Swimming inhibition assays of the six identified compounds. Gallic acid **(A)**, Catechin **(B)**, Ellagic acid **(C)**, Chlorogenic acid **(D)**, Quercetin **(E)**, and Kaempferol **(F)**.

Figure 9 shows that all six identified compounds could inhibit the swarming motility of *P. aeruginosa* PAO1. Among the six identified compounds, ellagic acid had the most remarkable inhibitory effect on swarming motility; at 0.1 mg/mL, the average swarming diameter of ellagic acid was 10.857 ± 1.309 mm, with no bacterial colony tendrils observed, which was significantly (*p* < 0.05) lower than that of the control. As shown in Table 4, the IC_50_ value of ellagic acid on swarming motility was 0.024 ± 0.008 mg/mL, whereas the IC_50_ values were 0.217 ± 0.018, 0.051 ± 0.006, 0.116 ± 0.014, and 0.037 ± 0.002 mg/mL for gallic acid, catechin, chlorogenic acid, and kaempferol, respectively, which were all higher than that of ellagic acid. In addition, the IC_50_ of quercetin on swarming motility was not detected because at the four tested concentrations, the inhibiting values all were higher than 50%, but lower than that of ellagic acid. Similarly, all six identified compounds showed inhibitory effects on *P. aeruginosa* PAO1 swimming motility (Figure 10). Interestingly, the inhibitory effect of ellagic acid on swimming motility was the strongest among the identified six compounds, with average bacterial colony diameters significantly (*p* < 0.05) decreased compared with the control. At 0.1 mg/mL, the average bacterial colony diameter of ellagic acid was 12.754 ± 1.004 mm, which was significantly (*p* < 0.05) lower that of the control (42.333 ± 2.517 mm). In addition, the IC_50_ value of ellagic acid on swimming motility (0.020 ± 0.003 mg/mL) was the lowest among the six compounds, (Table 4). These results indicate that ellagic acid is a remarkable inhibitor for swarming and swimming motility of *P. aeruginosa* PAO1, and might be the main active constituent of the DF to inhibit swarming and swimming motility of *P. aeruginosa* PAO1.

Our findings are supported by previous studies. Among the six identified compounds, catechin, ellagic acid, quercetin, and kaempferol have been reported to reduce the virulence factors of *P. aeruginosa* PAO1 (Singh et al., 2009; Vandeputte et al., 2010; Sarabhai et al., 2013; Ouyang et al., 2016), and chlorogenic acid and gallic acid in *Rosa rugosa* and *Moringa oleifera* have also shown inhibitory effects on QS-controlled phenotypes, indicating that all identified compounds show anti-quorum sensing potential (Singh et al., 2009; Zhang et al., 2014). Thus, these six compounds contributed to the inhibitory effects on pyocyanin production, swarming motility, and swimming motility of *P. aeruginosa* PAO1 in the DF of *C. nitidissima* Chi flowers.

In conclusion, to the best of our knowledge, this is the first study to report on the inhibitory effects of *C. nitidissima* Chi flower fractions on pyocyanin production, swarming motility, and swimming motility of *P. aeruginosa* PAO1 at sub-MICs. The *C. nitidissima* Chi fractions, especially the DF, showed inhibitory effects on pyocyanin production without influencing the growth of *P. aeruginosa* PAO1. The DF also inhibited swarming and swimming motility of *P. aeruginosa* PAO1 in a concentration-dependent manner. In addition, the DF significantly down-regulated the expressions of *lasR* (*p* < 0.05) and *rhlR* (*p* < 0.01) in *P. aeruginosa* PAO1 to cause the inhibitory effects on pyocyanin production, swarming motility, and swimming motility. We identified six compounds from the DF. All six identified compounds, especially ellagic acid, reduced the pyocyanin production, swarming motility, and swimming motility of *P. aeruginosa* PAO1. Thus, the inhibitory effects on the QS-controlled phenotypes of *P. aeruginosa* PAO1 might be attributable to these six and/or other compounds in the DF of *C. nitidissima* Chi flowers. Thus, the *C. nitidissima* Chi flower, especially the DF, might be a potential quorum sensing inhibitor of *P. aeruginosa* PAO1.

## Author contributions

RY and AJ conceived and designed the experiments. RY, YG, JZ, and ZH performed the experiments. RY, BS, ZW, and HC analyzed the data. RY and AJ wrote the paper.

### Conflict of interest statement

The authors declare that the research was conducted in the absence of any commercial or financial relationships that could be construed as a potential conflict of interest.
